# The importance of endobag use with incision-protective devices in VATS lung resection: a preliminary study

**DOI:** 10.1186/s13104-022-06047-7

**Published:** 2022-05-10

**Authors:** Carlos Andrés Latorre Noguera, Agnaldo José Lopes, Ivan Mathias Filho, Claudio Higa, Rodolfo Acatauassú Nunes, Carlos Eduardo Teixeira Lima, Eduardo Haruo Saito

**Affiliations:** 1 Pedro Ernesto University Hospital, State University of Rio de Janeiro (UERJ), Boulevard 28 de Setembro, 77, Vila Isabel, Rio de Janeiro, 20551-030 Brazil; 2 Medical Sciences Post-Graduation Program, School of Medical Sciences, State University of Rio de Janeiro (UERJ), Av. Prof. Manoel de Abreu, 444, 2º andar, Vila Isabel, Rio de Janeiro, 20550-170 Brazil; 3 Hospital Quinta D’Or, Rua Almirante Baltazar, 435, São Cristóvão, Rio de Janeiro, 20941-150 Brazil

**Keywords:** Pulmonary resection, Video-assisted thoracoscopic surgery, Endobag fluid

## Abstract

**Objective:**

The advent of new techniques such as video-assisted thoracoscopic surgery (VATS) for the removal of lung segments leads to compression of the surgical specimen, with the possible dissemination of neoplastic cells. The sheer volume of surgeries performed using these techniques has caused many institutions to stop removing the surgical specimen using an endobag, even when retractors/protectors are used in the instrumentalization incision. This study aimed to collect data from patients undergoing lung resection by VATS and analyze the cytopathological results of the collected material.

**Results:**

A total of 47 endobag fluid samples were collected from patients who underwent VATS. The surgical specimen was subjected to histopathological analysis, and all patients underwent pathological TNM staging. In the cytopathological analyses, only 2 (4.3%) specimens of endobag fluid aspirate were positive for neoplastic cells. In these two cases, the tumors were peripheral, both with diagnoses of moderately differentiated adenocarcinoma and with classifications of T1bN0M0 and T3N0M0. These results indicate that although there is a low incidence of tumor cells in endobag fluid, it is always better to perform surgery using all available protective measures to avoid tumor implantation in the thoracic cavity to the greatest extent possible.

## Introduction

Notable changes in the epidemiology and prevention of lung cancer have occurred in the last decade due to changes in smoking habits, advances in the understanding of tumor genetics and the role of the immune system in cancer control and new treatment options [1–4]. Despite these advances, lung cancer remains the leading cause of cancer death worldwide [5, 6]. Since 1985, lung cancer has been the leading cause of mortality worldwide, and approximately 13% of all new cancer cases are lung cancer [7]. The incidence rate has been decreasing since the mid-1980s among men and since the mid-2000s among women, possibly due to changes in smoking cessation behavior [7]. The relative 5-year survival rate for lung cancer is 18%, and only 16% of these cancers are diagnosed at an early stage, in which case the 5-year survival rate is 56% [6, 7].

In the current state of the art, lung cancer can be diagnosed histopathologically with sputum cytology, thoracentesis, accessible lymph node biopsy, bronchoscopy, transthoracic needle puncture, biopsy by video-assisted thoracoscopic surgery (VATS) or thoracotomy [8–11]. The initial evaluation of metastatic disease depends on the patient's history and the results of the physical examination, laboratory tests, chest computed tomography and positron emission tomography, as well as tissue confirmation if there is suspected mediastinal involvement. The need for an additional evaluation for metastases depends on the clinical presentation [8, 12]. Treatment and prognosis are closely linked to the histological type and tumor staging. For non-small-cell carcinomas at stages I to IIIA, surgical resection is preferred [13, 14]. Advanced non-small-cell carcinoma is treated with a multimodal approach that may include radiotherapy, chemotherapy, target cell therapy, immunotherapy and palliative care [15].

In contemporary VATS lobectomy or segmentectomy, the essential characteristic is that the view is achieved and exhibited using video technology and not direct vision. The need for some type of utilitarian incision to allow the removal of the surgical specimen gives the technique different requirements than diagnostic pleuroscopy. In VATS, there is no need to maintain carbon dioxide inflation without intracavitary leakage, as is necessary for laparoscopy. In the thorax, the lungs collapse, and the ribs maintain the space. A large incision is necessary to remove the surgical specimen containing the tumor; this incision allows the insertion of the optical device and some conventional surgical instruments, and one or two trocars can be used, depending on the selected technique [16–20]. The sheer volume of surgeries performed using VATS has caused many institutions to stop removing the surgical specimen into an endobag when retractors/protectors are used for the instrumentalization incision. The objective of this study was to collect data from patients undergoing lobectomy surgery or pulmonary segmentectomy by VATS and to analyze the cytopathological results of the collected material—either aspirated from the liquid or washed from the endobag—in order to determine whether it is necessary to use an endobag when using retractors/protectors for the instrumentalization incision.

## Main text

### Methods

**Fig. 1:**
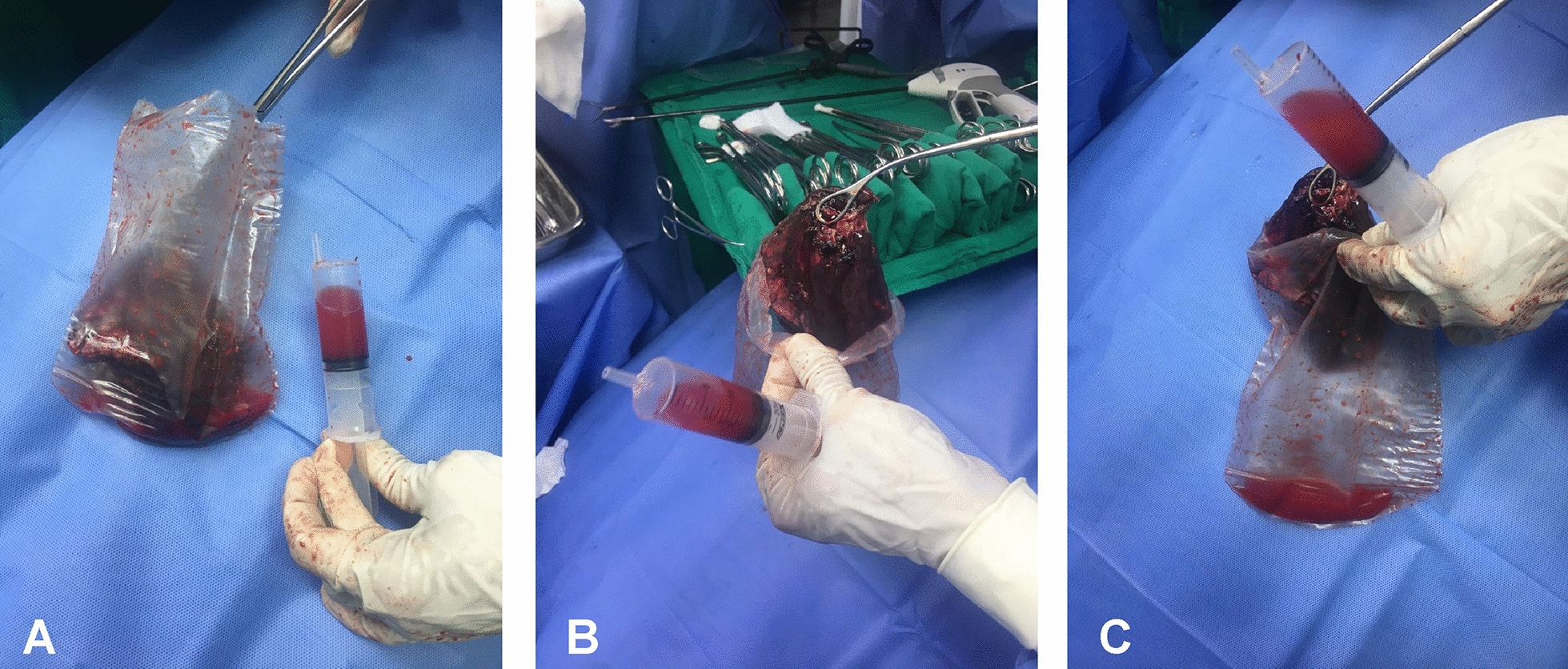
The endobag fluid was aspirated for cytopathological analysis (**A**). Then, the surgical specimen was removed from the endobag to determine whether more liquid had been collected (**B**). Finally, when the surgical specimen was removed from the endobag, more liquid was aspirated for cytopathological analysis (**C**)

This was a preliminary retrospective study that included 47 consecutive patients with lung injury who underwent thoracic surgery (lobectomy or pulmonary segmentectomy) via VATS between January 2020 and February 2021 at the Department of Thoracic Surgery, Pedro Ernesto University Hospital, State University of Rio de Janeiro (UERJ), Rio de Janeiro, Brazil. All of the safety protocols that this type of intervention requires were followed, including the use of surgical incision protectors and the extraction of the surgical specimen into an endobag device (Fig. 1). The extracted material was sent for histopathological analysis and tumor-node-metastasis (TNM) staging, and liquid samples, including both liquid aspirate and surgical specimens from the endobag, were submitted to cytopathological analysis.

This study was approved by the Research Ethics Committee of the Pedro Ernesto University Hospital, UERJ, Rio de Janeiro, Brazil, under number CAAE-48443921.2.0000.5259.

### Results

The 47 study participants included 35 women and 12 men with a mean age of 60 ± 11 years. The general clinical characteristics and pulmonary function parameters in the preoperative period are shown in Table 1. On computed tomography of the chest, the following anatomical distribution of neoplasms was observed: peripheral lesions in 41 (87.2%) participants and central lesions in 6 (12.8%) participants.

**Table 1:** General clinical characteristics and pulmonary function in the preoperative period

Variable	Value
Demographic data	
Age (years)	60 ± 11
Gender (female)	35 (74.5%)
Weight (kg)	66 ± 6.3
Height (cm)	167 ± 8
BMI (kg/m^2^)	23.7 ± 4.5
Smoking history	38 (80.9%)
Comorbidities	
Chronic obstructive pulmonary disease	16 (34%)
Systemic hypertension	15 (31.9%)
Diabetes mellitus	6 (12.8%)
Dyslipidemia	5 (10.6%)
Pulmonary function	
FEV_1_ (% predicted)	69.5 ± 13.7
FVC (% predicted)	81 ± 14.5
FEV_1_/FVC (%)	73 ± 7
DLCO (% predicted)	58 ± 11

The results are expressed as the mean ± SD or a number (%)

*BMI* Body mass index, *FEV*_*1*_ Forced expiratory volume in 1 s, *FVC* Forced vital capacity, *DLCO* Diffusing capacity for carbon monoxide

**Fig. 2:**
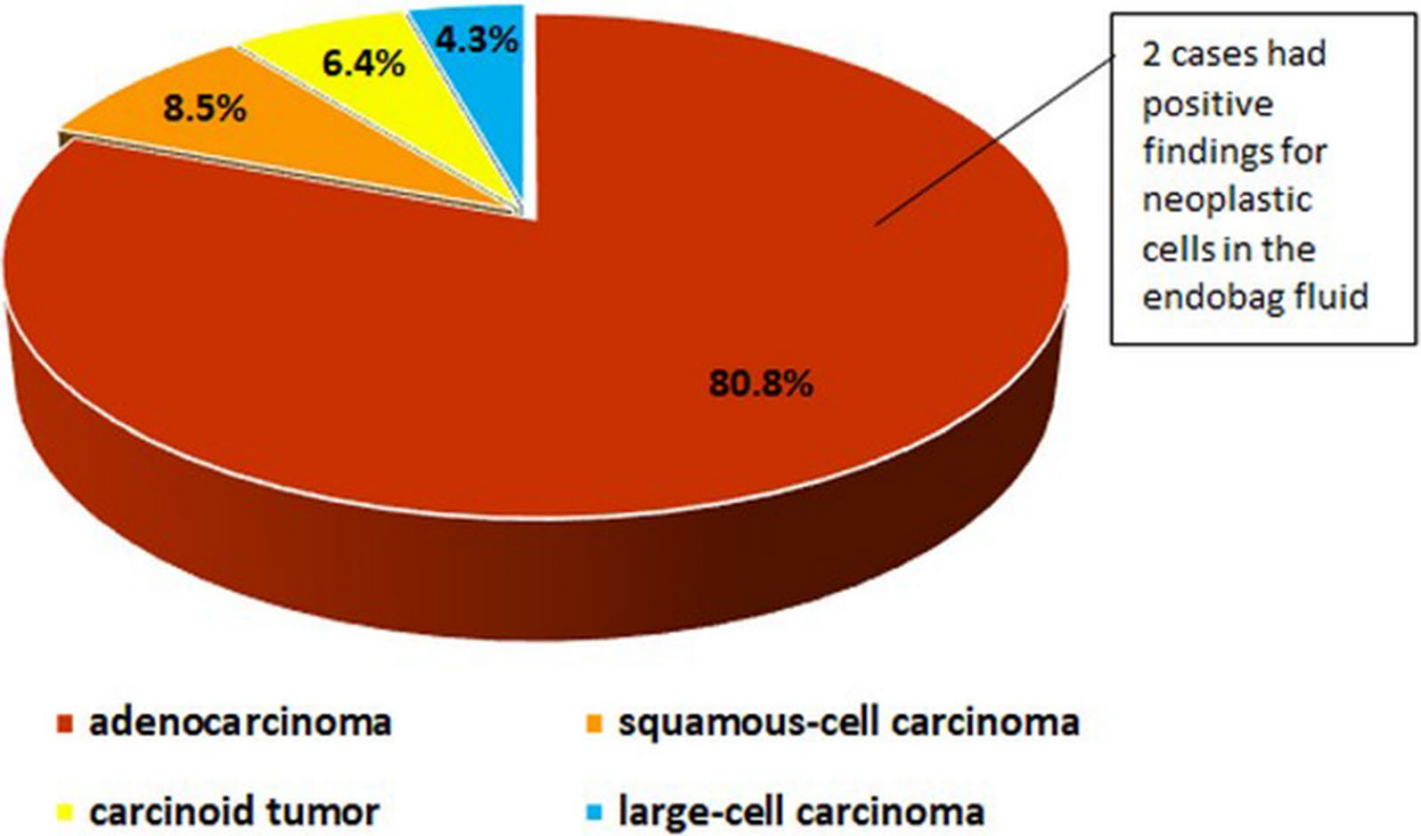
Distribution of cases according to histopathological type in evaluated sample

Regarding histopathology, there was a clear predominance of adenocarcinomas; less frequently, squamous-cell carcinomas, carcinoid tumors, and large-cell carcinomas were observed. The distribution of cases according to histopathological type is shown in Fig. 2. Of all of the evaluated participants, only 2 (4.3%) had positive findings for neoplastic cells in the endobag fluid; both were diagnosed with moderately differentiated adenocarcinoma. Regarding TNM staging, one of the cases that was positive for malignancy was classified as T1bN0M0, while the other was classified as T3N0M0.

### Discussion

Lung cancers are categorized as small-cell carcinomas or non-small-cell carcinomas, and the latter are subdivided into squamous-cell carcinomas (25–30% of cases), adenocarcinomas (40% of cases) and large-cell carcinomas (10–15% of cases) [21–23]. In addition to these types, other tumors that can reach the lungs include carcinoid tumors (fewer than 5% of cases), adenoid cystic carcinomas, lymphomas and sarcomas [22]. These categories are used to inform the treatment decision and determine the prognosis [23–25]. Signs and symptoms may vary depending on the type of tumor and the extent of the metastases. The diagnostic evaluation of patients with suspected lung cancer includes histopathological diagnosis; staging, including the evaluation of metastases; and functional evaluation of the patient for pulmonary resection surgery [23, 26]. In this study, unlike others [21–23], the vast majority of cases were adenocarcinomas (80.8%).

New surgical techniques, such as VATS, require measures to protect the surgical incision, such as retractors or protectors, to prevent neoplastic cells from implanting in the surgical wound due to the contact of surgical instruments with the incision tissues; furthermore, a protective bag should be used at the time of removal of the surgical specimen, particularly if it is a lobe or pulmonary segment [16–20]. The implantation of neoplastic cells in the chest wall after VATS lung surgery is rare. In the current study, the results of patients who were undergoing pulmonary lobectomy by VATS were investigated, regardless of the involved pulmonary lobe or the location of the lesion (peripheral or central), using an incision protector and an endobag to remove the surgical specimen. Specifically, the cytopathological results of the endobag fluid were analyzed. The incidence of neoplastic cells inside the endobag when the specimen was removed was low (less than 5%). However, it is still important to use an endobag for the removal of the surgical specimen in this type of surgery because of the possibility of neoplastic cell implantation in the thoracic cavity.

There are documented protection options in addition to the use of endobags, such as washing the thoracic cavity with serum after the resection and removal of the surgical specimen. All of these procedures have been described as effective for preventing tumor implants in the chest wall after pulmonary lobectomy [27–29]. However, there are documented cases of implantation in the chest wall after the VATS resection of tumors with specific diagnoses, such as thymoma; thus, VATS should be performed with extreme caution for these tumors because there are multiple cases of contamination metastasis reported in the literature [30, 31].

In conclusion, the findings of the present study indicate that although there is a low incidence of neoplastic cells in endobag fluid, endobag use should continue when retractors/protectors are used for the instrumentalization incision during VATS. This is because it is always best to perform surgery using all available protective measures to avoid implantation of neoplastic cells in the thoracic cavity to the greatest extent possible.

## Limitations

One of the main limitations of this study was the small sample size. Another limitation that should be highlighted was the nonstandardization of the T descriptor, which ranged from T1b to T3. This lack of standardization could interfere with the results, especially in larger tumors. In fact, the size of the tumor is more important than its location since during the removal of surgical specimens, larger tumors undergo greater compression, regardless of the central or peripheral location of the lesion. Another issue is the need to evaluate patients with tumors of any size that invade the visceral pleura, as these tumors may be more likely to disseminate neoplastic cells when the surgical specimen is removed.

## Data Availability

Data and materials are available from the corresponding author upon reasonable request.

## Abbreviations

BMI: Body mass index
DLCO: Diffusing capacity for carbon monoxide
FEV_1_: Forced expiratory volume in 1 s
FVC: Forced vital capacity
TNM: Tumor-Node-Metastasis
UERJ: State University of Rio de Janeiro
VATS: Video-assisted thoracoscopic surgery
